# Application of *In-Situ* and Soft-Sensors for Estimation of Recombinant *P. pastoris* GS115 Biomass Concentration: A Case Analysis of HBcAg (Mut^+^) and HBsAg (Mut^S^) Production Processes under Varying Conditions

**DOI:** 10.3390/s21041268

**Published:** 2021-02-10

**Authors:** Oskars Grigs, Emils Bolmanis, Vytautas Galvanauskas

**Affiliations:** 1 Laboratory of Bioprocess Engineering, Latvian State Institute of Wood Chemistry, LV-1006 Riga, Latvia; emils.bolmanis@biomed.lu.lv; 2 Latvian Biomedical Research and Study Centre, LV-1067 Riga, Latvia; 3 Department of Automation, Kaunas University of Technology, LT-51367 Kaunas, Lithuania; vytautas.galvanauskas@ktu.lt

**Keywords:** biomass concentration, *in-situ* and soft-sensors, turbidity, permittivity, signal filtering, off-gas analysis, stirred-tank bioreactor, *Pichia pastoris*

## Abstract

Microbial biomass concentration is a key bioprocess parameter, estimated using various labor, operator and process cross-sensitive techniques, analyzed in a broad context and therefore the subject of correct interpretation. In this paper, the authors present the results of *P. pastoris* cell density estimation based on off-line (optical density, wet/dry cell weight concentration), *in-situ* (turbidity, permittivity), and soft-sensor (off-gas O_2_/CO_2_, alkali consumption) techniques. Cultivations were performed in a 5 L oxygen-enriched stirred tank bioreactor. The experimental plan determined varying aeration rates/levels, glycerol or methanol substrates, residual methanol levels, and temperature. In total, results from 13 up to 150 g (dry cell weight)/L cultivation runs were analyzed. Linear and exponential correlation models were identified for the turbidity sensor signal and dry cell weight concentration (DCW). Evaluated linear correlation between permittivity and DCW in the glycerol consumption phase (<60 g/L) and medium (for Mut^+^ strain) to significant (for Mut^S^ strain) linearity decline for methanol consumption phase. DCW and permittivity-based biomass estimates used for soft-sensor parameters identification. Dataset consisting from 4 Mut^+^ strain cultivation experiments used for estimation quality (expressed in NRMSE) comparison for turbidity-based (8%), permittivity-based (11%), O_2_ uptake-based (10%), CO_2_ production-based (13%), and alkali consumption-based (8%) biomass estimates. Additionally, the authors present a novel solution (algorithm) for uncommon *in-situ* turbidity and permittivity sensor signal shift (caused by the intensive stirrer rate change and antifoam agent addition) on-line identification and minimization. The sensor signal filtering method leads to about 5-fold and 2-fold minimized biomass estimate drifts for turbidity- and permittivity-based biomass estimates, respectively.

## 1. Introduction

*Pichia pastoris* (*Komagataella pastoris*) is a yeast culture widely used in biotechnology and is capable of expressing various types of recombinant proteins, under submerged bioreactor cultivation conditions, that are of major importance [1,2]. Hepatitis B core- (HBcAg) [3,4] and surface- (HBsAg) [5,6] antigens are recombinant protein examples being investigated for improved vaccines and agents used in biomedicine development. Hepatitis B antigens can be also used in biosensing development for clinical assays [1]. Under optimal process conditions, the process productivity depends on the overall number of microorganisms and fraction of those which are in active target product production-, metabolic- or propagation-state [7]. Correct and well-interpreted information on actual total or viable cell mass concentration therefore is key bioprocess information. Moreover, minimal requirements for real-time process supervision is of utmost importance when concerning pharmaceuticals production processes that are subject to GMP guidelines [8].

Literally microbial biomass concentration is expressed as a mass or number of cells attributed to liquid sample volume (e.g., g/L, kg/m^3^, cells/mL, cells/m^3^, etc.). Common direct off-line biomass concentration analysis methods include gravimetric dry/wet cell weight measurements and cell counting with chemocytometers [9], Coulter counters [10] and flow-cytometers [11]. These direct analyses are usually calibrated to instrumental methods to speed up the biomass measurement, improve reliability, and for automation purposes. These instrumental methods utilize physical (optical and electrical), chemical (interactions with enzymes, dyes, antibodies, etc.) or physiological (e.g., O_2_ consumption, CO_2_ production, acidification) characteristics of the analyzed sample or cultivated culture [12,13,14]. For *in-situ* and on-line applications the most popular are optical density (OD) (known also as turbidity), specific capacitance (detected by dielectric spectroscopy) and various soft-sensor (based on culture physiology related variables) measurement use in estimations described in detail further. *In-situ* microscopy has also proven its applicability in on-line biomass concentration monitoring [15].

Industrially, the most common method employed for biomass detection is based on the interaction between culture and light. There are three different basic effects of interactions between culture and light used in biomass OD detection methods: absorption, emission, and scattering [14]. Mathematical expressions of various forms (e.g., linear [16,17] polynomial [16], power functions, etc.) are used for OD or turbidity measurement correlation to biomass concentration. The typical wavelength range includes the light from ultraviolet (UV) to mid-infrared (MIR) spectrum. UV to near infrared (NIR) light (350–1100 nm) is used for culture OD measurement with photometers [18], *in-situ* [19] or at-line [20,21] instruments. Lack of UV and low wavelength visible spectrum (VIS) light for OD measurement is it cross-sensitivity with other components present in the culture broth [22]. Majority of OD NIR *in-situ* sensors (turbidimeters) use light wavelengths of 800–1100 nm, because most culture media absorb very little light in this region [12]. Usually, low wavelength *in-situ* NIR systems are used for accurate biomass estimation up to 20–40 DCW [12], however, under constant mixing and aeration conditions, good quality biomass estimates of up to 90 DCW have been reported [23]. There are no extensive studies, in which high cell density biomass (>90 DCW) is monitored using a turbidity probe with acceptable or identified accuracy. In comparison to the *in-situ* turbidity method, current off-line and *in-situ* microscopy-based techniques are limited for quantification and morphological analysis of relatively large cells, such as microalgae [24] and yeast [15,25], and have been employed for cell densities up to just 60–70 yeast DCW.

Dielectric properties of the cell suspension, such as capacitance (pF) and permittivity (pF/cm), proportionally relate to viable cell volume. This phenomenon is used by *in-situ* permittivity sensors [26,27] and Coulter-counters [28,29]. Modern instruments use frequency-scanning techniques in a range from 100 KHz to 20 MHz, capable for cell size and biomass concentration estimation (dielectric spectroscopy). Reported measurement ranges are up to 35–155 DCW [26,30] in high cell density cultures. The reported method demonstrates limited applicability in low biomass (<~5–10 g/L [29,30]) and high conductivity conditions [12,13]. In the reported research, linear [26,29] or mixed linear-nonlinear [29] relationships between DCW and permittivity can be observed for various bacterial and yeast cultures cultivated under varying conditions. As the permittivity measurement selectively accounts for the viable cell volume (compared to DCW, in which also nonviable cells are counted), it is obvious that under varying process conditions the correlation between permittivity and DCW may differ. This can be especially true for the methanol induction phase in methylotrophic yeast *P. pastoris* cultivations, in which variations in cell viability and size are present at different growth rates [31]. In this case, alternatively to the DCW measurement, generally accepted as the standard biomass measure reference, evaluation of soft-sensor performance, if calibration is made to the reference permittivity-based biomass estimates, is an interesting aspect not widely investigated elsewhere.

A common characteristic of *in-situ* turbidity and permittivity measurements is a cross-sensitivity to the stirring rate, bubble size and their movement near/trough the sensor-measuring tip that were observed or discussed in probe application reports published elsewhere [18,32,33,34,35]. To avoid this uncommon side effect, degassed chambers or signal filtering is suggested. Typically such uncommon *in-situ* turbidity and permittivity sensor shift amplitudes are too high to be avoided or minimized by simple moving average or Savitzky–Golay filtering algorithms [33] usually useful for smoothing random measurement noise. Instead, the use of methods that validate the sensor signal reading prior to its use for biomass estimation are necessary. A method for *in-situ* turbidity sensor signal stabilization (validation) using swarm intelligence is applicable [36], however, this technique requires high implementation efforts and additional process data, like base feed, off-gas CO_2_ and O_2_, and DO use and analysis. Alternatively, to the previous method, there is a lack of examples using a simple rate analysis of the *in-situ* sensor signal for measurement validation and faulty shift exclusion.

Process-data and model-based soft-sensor methods, in combination or used separately, have been proven for application in biomass estimation [12,36]. Data driven methods using measures of biomass physiological activity, such as O_2_ consumption/CO_2_ production [37,38,39], alkali amount added for compensation of acidification [37,38,40] are the most popular approaches. Typical soft sensors are mathematical models based on growth kinetics (data driven) or statistical analysis (such as multilinear regression (MLR) or principal component analysis (PCA)), neural networks, or combinations of all of these techniques [12,37]. Process data- and model-based estimates can also be used in Kalman [37,41] and particle [41] filters for improved estimation accuracy, however these techniques are complex and, therefore, difficult to implement.

The bottleneck for the soft-sensor performance is the accuracy of conversion rate calculation. While random (measurement noise) errors can be minimized quite easily with data smoothing algorithms, this is not the case for systematic errors caused by miscalibrations, inaccuracy of analytical devices or various technical shortcomings of the biomass related measuring sensor [42]. Moreover, as it was shown for the *P. pastoris* culture [39,43], yield coefficients are not constant for different biomass growth rates. As proposed elsewhere [44,45], performance of off-gas biomass estimators may also be influenced by badly tuned dissolved oxygen (DO) control followed by significant fluctuations in DO-related variables, i.e., off-gas data of CO_2_ and O_2_. Therefore, systematic errors can only be detected and possibly reduced by making use of all available information in terms of the first-principle (elemental balancing) constraints and the accuracy of turnover rates in reconciliation procedures, what are well described elsewhere [39,46,47].

Characteristic biomass concentrations achieved at the end of *P. pastoris* high-cell-density cultivation processes are 100–185 DCW [17,30,35,48]. In most of the cultivations, glycerol and methanol substrates are fed consecutively. Several stress factors, influencing biomass viability and morphology of HBsAg producing *P. pastoris* Mut^S^ strain were reported [49]. In comparison to the Mut^S^ strain, *P. pastoris* Mut^+^ strain, is more tolerant to methanol, what would possibly lead to higher viability indicators, e. g., permittivity, compared to the Mut^S^ strain. At the beginning of the methanol fed-batch phase, culture metabolism adapts to toxic methanol and the induction of recombinant protein synthesis. High residual methanol levels (>5–10 g/L) are toxic for cells [50,51]. Therefore, different residual methanol and oxygen levels may influence biomass optical, dielectric and physiologic conditions used for biomass quantification with the methods introduced previously. Just a few research articles are available (one of them from Godfeld et al. [30]) where high cell density (≥90 DCW) *P. pastoris* biomass detection with solid at-line, *in-situ* or soft-sensor-based biomass estimation methods are analyzed for a rather high number of experiments. Therefore, from the information available in the scientific or practical application reports, it is often difficult to evaluate method applicability under ‘real process’ conditions with the reviewed process disturbances and cross sensitivity to parameters like anti-foam agent addition or rapid stirrer rate change influence on biomass estimation accuracy.

Application of various standard off-line (OD/WCW/DCW), *in-situ* (turbidity/permittivity) and soft-sensor-based (off-gas O_2_/CO_2_; alkali consumption) methods for 13 high cell density *P. pastoris* cultivations is presented in this contribution. For the different process stages and biomass levels, simple approximation models were identified and used. Biomass estimation results, in the context of varying process conditions, were analyzed. Aspects of the practical implementation and interpretation of the applied methods are discussed. A practical example for the oxygen uptake rate calculation for the bioprocess with oxygen enrichment and one O_2_ off-gas analyzer is demonstrated. Finally, a novel solution (algorithm) for uncommon *in-situ* turbidity and permittivity sensor signal shifting, caused by intensive stirrer rate change and antifoam agent addition, was implemented and tested experimentally.

## 2. Materials and Methods

### 2.1. Cultivation Conditions

Cultivation of both HBcAg (Mut^+^) and HBsAg (Mut^S^) recombinant *Pichia pastoris* GS115 strains was performed in a series of experiments in a 5 L fully automated bench-top bioreactor system EDF-5.4 (Biotehniskais Centrs AS, Riga, Latvia). In general, cultivation conditions corresponded to the Invitrogen corporation cultivation guidelines for Mut^+^ and Mut^S^ strains, respectively. However, some parameters (see Table 1) varied due to recombinant protein production screening or technical reasons, namely, residual methanol levels (0.01–5 g/L) during the protein production phase, process temperature (throughout the whole process) (30 ± 0.1 or 24 ± 0.1 °C), dissolved oxygen (DO) level (1–40%) and aeration rate (1.7 or 3.0 slpm). 

Additional Mut^S^ strain cultivation under conditions proposed by Gurramkonda et al. [50] was performed, and two different residual methanol set-points 2 and 6 g/L were tested. The main differences between the Invitrogen’s and Gurramkonda’s protocols were related to the DO and excess methanol levels, as well as some differences in the batch and fed-batch media nutritional content.

Before the start of the cultivation process, the culture medium pH was adjusted to 5.0 ± 0.1 (Invitrogen protocol) or 5.6 ± 0.1 (Gurramkonda’s protocol) using a 28% NH_4_OH solution, which was also used to control the set pH value during the cultivation (peristaltic pump: WP10-S 3/16 L4-B, Welco, Tokyo, Japan; tubing: inner diameter 3.2 mm, outer diameter 6.4 mm). DO set-point of 30 ± 5% was controlled by automatically adjusting the stirrer rotational speed (200–1000 rpm) or additional inlet air enrichment with O_2_ regarding Table 1.

As it is indicated in the Table 1, in some experiments DO level insignificantly differed from the set-point. The aeration and O_2_ enrichment procedure is described in the further subsections of this chapter. An outlet gas condenser was used for humidity condensing from the exhaust gas to minimize evaporation and the water content in the off-gas. Off-gas drying through glycerol concentrate and silica gel was used. The foam level was controlled by the addition of antifoam 204 (Sigma).

Four datasets characterizing a set of experiments to be calibrated to different models or set of experiments, in which biomass measurements are available for specific method or process comparison, are introduced. Dataset 1 (experiments 3c, 4c, 5c, 6c, 1s, 2s, 4s, 5s, 6s and 7s) and Dataset 2 (experiments 1c and 2c) are used for different calibration model evaluation for the turbidity measurement. Dataset 3 (experiments 3c, 4c, 5c and 6c) forms a set of 4 experiments from which the performance of used *in-situ* turbidity/permittivity and soft-sensor techniques can be compared for the case of using constant soft-sensor parameters. Finally, Dataset 4 (experiments 1s, 2s, 3s and 6s) consists of Mut^S^ strain cultivation experiments with permittivity measurement available as well.

**Figure 1:**
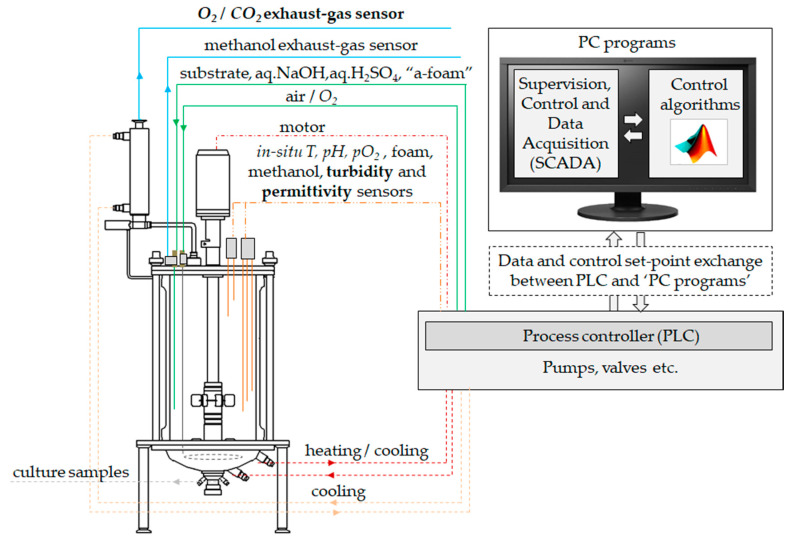
Schematic diagram of the bioreactor and controls architecture.

The bioreactor setup consists of a glass vessel and a stainless steel upper and bottom lid (see Figure 1). The reactor has a working volume of 2–4 L, two standard Rushton turbines, and an outlet gas condenser. The process controller (PLC) has 3 DI/DO, 4 AI/AO and 1 relay input unit (Siemens AG, Germany). The process analytical tools of the off-gas O_2_ and CO_2_ measurement (Bluesens, Herten, Germany; BlueInOneFerm; measurement ranges for O_2_ and CO_2_, respectively, were up to 50 and 25 vol.%), culture turbidity (ASD19-EB-01, Optek, Essen, Germany; light absorption (transmission) measurement within 840–910 nm wavelength range; optical path length 10 mm) and permittivity (Hamilton, Bonaduz, Switzerland, Incyte) were connected to the PLC and utilized for process monitoring. PC (SCADA) was connected to the PLC through a router via Ethernet link. Programming in Matlab (R2019a, Mathworks, Natick, MA, USA) .m code was used for implementation of the proposed biomass estimation and on-line sensor filtering algorithms. The data exchange of the process and control variables between the control algorithms (Matlab) and SCADA (programmed in the software platform of PcVue Solutions, Ltd., Sèvres, France) was implemented every 1 s through an OPC server. Detailed information on the reactor vessel and control system configuration can be found in the research published earlier [52].

### 2.2. Off-Line Measurements

Cell growth was monitored by off-line measurements of the optical density (OD) at a wavelength of 590 nm (GRANAT, KFK-2, St. Petersburg, Russia). Wet cell weight (WCW) and dry cell weight (DCW) concentration measurements were determined gravimetrically. Biomass samples were placed in pre-weighted Eppendorf tubes^®^ and centrifuged at 13,200 RPM for 5 min. Afterwards, the supernatant was discarded, and the cells were resuspended in distilled water and centrifuged once more. The liquid phase was discarded, and the remaining wet cell biomass was weighted. Afterwards, the samples were dried at 105 °C until a constant weight was reached, and the dry cell biomass was determined. 

Off-line methanol was measured using gas chromatography (6890 N GC Agilent, Santa Clara, CA, USA). 

All yield parameters calculated attributing them to dry cell weights.

### 2.3. Turbidity and Permitivitty Signal Acquisition and Filtering

Turbidity sensor signal recording was made every 60 s. Signal preprocessing parameters were chosen to provide stable sensor readings (symmetric signal damping and 60 s as an integration time for signal damping). Permittivity was measured and calculated by the Incyte ‘Frequency Scan’ mode with 17 simultaneous measurements across a frequency range of 0.3–10 MHz. Permittivity measurements were made every 2 s and integrated, creating a moving average over a defined period. These acquisition periods varied in experiments 3c, 4c, 5c, 6c, 1s, 2s, 3s, and 4s; their respective values in seconds were 60, 60, 60, 60, 60, 720, 360, and 60.

**Figure 2:**
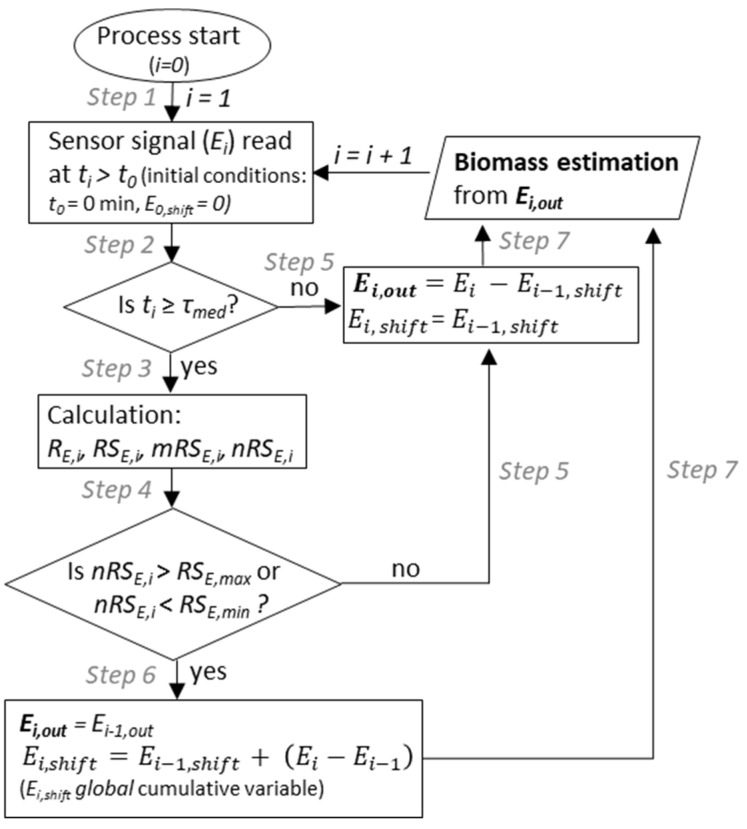
Flow chart of the on-line turbidity and permittivity signal filter algorithm.

The turbidity and permittivity signal filtering technique was implemented for sudden signal jumps and drops initiated by sudden stirrer rotational speed changes and antifoam agent addition. The concept of the filtering method is based on sensor signal change rate analysis allowing to count rapid sensor signal shifts, which are uncharacteristic for common biomass growth or cell lysis behavior. Counting such uncommon sensor signal shifts allows for subtraction of accumulated shifts from the actual sensor readings. The filtering algorithm is presented in Figure 2.

Further the filtering algorithm main execution steps are described. The actual sensor signal is read (*E_i_*) at the time moment *t_i_* (Step 1). As the filter algorithm uses a median calculation over a time period of *τ_med_* = 30 min ahead of each iteration (procedure described further), execution of the main filtering loop could take place when the sensor signal sampling time or *t_process_* is equal or exceeds *τ_med_* (Step 2). Initially, in Step 3, the following parameters are calculated: time difference between the sensor signal sampling events (signal sampling frequency *Δt* = 1 min):

(1) $$\Delta t=t_{i}-t_{i-1}$$

sampled sensor signal difference:

(2) $$\Delta E=E_{i}-E_{i-1}$$

sensor signal change rate at the moment of a new sensor signal reading:

(3) $$R_{E,i}=\frac{\Delta E}{\Delta t}$$

sensor signal change rate sum at the moment of a new sensor signal reading:

(4) $${\mathrm{RS}}_{E,i}=\overset{i}{\underset{i-\tau_{sum}}{\sum }}R_{E,i}$$

where *τ_sum_* indicates the amount of the last RS_E_ samples to be summed if the sensor signal sampling frequency is 1 min. In this research, *τ_sum_* = 5 min was used for both turbidity and permittivity probe signal filtering. Summing of *R_E_* allows to obtain less noisy and extended height peaks of uncommon signal change rate, therefore it is easier to identify them. From the results presented in the Results section, it can be seen that explicit RS_E_ shifts from zero (RS_E_ zero-baseline shift) are observable for turbidity measurement within the process 10–30 h (for permittivity-based measurement, this deviation is practically negligible). The median of RS_E_ calculation (Equation (5)) and its subtraction from RS_E_, are performed for signal normalization (Step 3) to exclude the RS_E_ zero-baseline shift phenomenon. The median of the sensor signal change rate sum at the time moment *t_i_*:

(5) $${\mathrm{mRS}}_{E,i}=\mathrm{median}\left({\mathrm{RS}}_{E,i-\tau_{\mathrm{med}}}:{\mathrm{RS}}_{E,i}\right)$$

where *τ_med_* indicates the amount of the last RS_E_ samples to be used for RS_E_ median value calculation, if the sampling frequency is 1 min. In this research, *τ_med_* = 30 min was used for both turbidity and permittivity probe signal filtering. Normalized sensor signal change rate sum at the time moment *t_i_*:

(6) $${\mathrm{nRS}}_{E,i}={\mathrm{RS}}_{E,i}-{\mathrm{mRS}}_{E,i}$$

Normalized RS_E,i_ (nRS_E,i_) then is compared with the allowed preset minimum and maximum limit values of RS_E__,min_ and RS_E,__max_ (Step 4) (identified RS_E__,min_ and RS_E__,max_ shown in the Results section). If nRS_E_ exceeds the preset minimum or maximum bounds, then the algorithm considers the inappropriate behavior of the biomass concentration increment/decrement, and substitutes the sensor signal reading with the last output from the filter (*E_i,out_ = E_i-_*_1,*out*_) (Step 6). The filter accounts for the difference between the actual and previous raw sensor signal readings, and sums it to the cumulative shift parameter (*E_i,shift_*) that accounts for all the registered shifts from a particular process. *E_i,shift_* can be positive or negative and is subtracted from the raw *E_i_* (*E_i,out_ = E_i_ − E_i,shift_*) each time when no pre-set inappropriate behavior of the biomass concentration increment/decrement occurs (Step 5). *E_i,out_* is used in biomass estimation (Step 7).

### 2.4. Turbidity Signal Approximation to DCW

**Figure 3:**
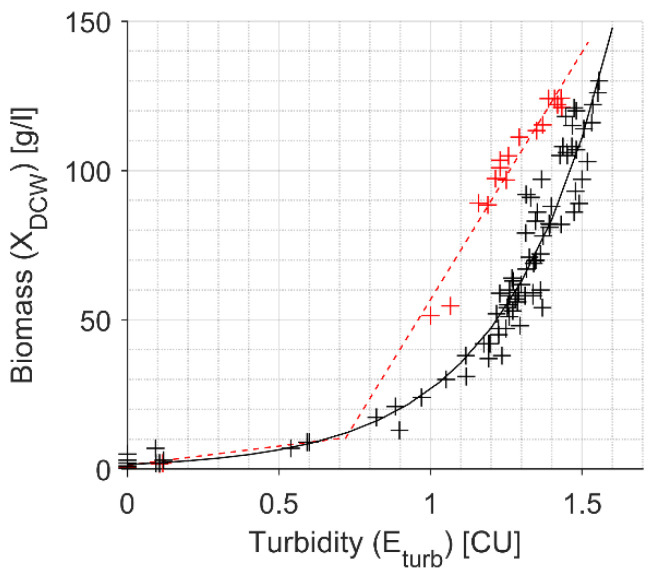
*In-situ* turbidity signal (*E_turb_*) approximation to DCW. Sparse points of the *E_turb_* from the processes with aeration *Qair* = 3.0 slpm (black plus, Dataset 1) and *Qair* = 1.7 slpm (red plus, Dataset 2); solid and dashed lines, respectively, represent the fitted models *Exponential 1* and *Linear* shown in Table 2.

Two datasets of experiments, Dataset 1 and Dataset 2, representing two aeration regimes 3.0 slpm and 1.7 slpm used respectively, were identified for different representation of DCW and *E_turb_* relationship in the high cell density region (calibration data and identified models shown in Figure 3 and Table 2 respectively).

For the Dataset 1 experiments, *Exponential 1* model:

(7) $$X_{turb}=a\cdot \exp(b\cdot E_{turb})$$

was identified, approximating DCW measurements in the whole turbidity measurement range of 0–1.55 CU. For the Dataset 2 experiments, *Exponential 2* model:

(8) $$X_{turb}=a\cdot \exp(b\cdot E_{turb})+c\cdot \exp(d\cdot E_{turb})$$

was identified, approximating DCW measurements in the turbidity measurement range of 0–1.40 CU. However, the model *Exponential 2* has a parabolic-like curvature with a narrow maximum within 1.40–1.45 CU, and a sharp spike. The same correlation quality for Dataset 2 was obtained by the *Linear* model:

(9) $$X_{turb}=a\cdot E_{turb}+b$$

for the two measurement ranges of *E_turb_* ≤ 0.72 and *E_turb_* > 0.72, each having its own set of model parameters. Identified *Exponential 1* and *Linear* models were used for *in-situ* turbidity-based biomass estimation for Dataset 1 and Dataset 2 experiments respectively, presented in the Results section.

### 2.5. Permittivity Signal Approximation to DCW

For biomass concentration (*X_perm_*) estimation from the permittivity signal (*E_perm_*), a linear relationship (Equation (10)) was used:

(10) $$X_{perm}=CF_{X}\cdot E_{perm}$$

Fitting the experimental permittivity signal data to off-line DCW measurements (Dataset 3 experiments 3c, 4c, 5c, 6c; and experiments 1s, 2s, 3s, 4s) from glycerol batch and fed-batch phases, when cell viability is close to 100%, the correlation between the *X_perm_* and CF_X_ parameters can be considered linear (calibration data enclosed in Appendix E, Figure A13). Experimental data showed that the pre-induction permittivity measurement correlation to DCW did not significantly differ for different experiments. The cell factor CF_X_ = 4.04 g/L/pF/cm was identified and used to calculate permittivity-based biomass concentration estimates for both glycerol and methanol consumption phases presented in this research. A similar approach to determine CF_X_ is presented by Horta et al. [29]; however, they analyzed this correlation for dry biomass values only up to approx. 7 g/L. After methanol induction, which can be accompanied by physiochemical or morphological changes in the cell [39], the *E_perm_* correlation to DCW is no longer linear. As it appears from the results shown further, in the methanol consumption phase, the use the same correlation cell factor identified for glycerol phase (4.04 g/L/pF) lead to a varying-quality fit for DCW measurements even for the experiments performed under similar conditions. The possible reasons for this phenomenon are discussed further.

### 2.6. OUR and CPR Calculation

Information from the culture oxygen uptake rate (OUR) and carbon dioxide production rate (CPR) can be used for biomass concentration quantification. In steady-state conditions, the oxygen uptake rate (OUR) can be assumed to be equal to the oxygen transfer rate (OTR), OUR = OTR. For online OUR and CPR calculation, information about O_2_ and CO_2_ concentrations in the bioreactor inlet and outlet gas lines, along with the gas flow rates in these lines, is required. The estimation precision depends on identification and the control precision of the previously mentioned parameter. In the majority of the conducted experiments, inlet air enrichment with O_2_ was used to follow the guidance from Invitrogen Co. cultivation protocols, according to the requirement of the relatively high dissolved oxygen (DO) level control. Air and O_2_ enrichment flows were controlled with separate rotameters and automatic valves for both gases (system configuration enclosed in Appendix C, Figure A7). Extra oxygen was added by the means of oxygen pulses. During these oxygen pulses (oxygen valve ‘open’) the air valve is closed simultaneously. At the end of oxygen pulses, (oxygen valve ‘close’), the air valve opens. The inlet gas flow rate was organized to have the same rotameter set-point for both gases (*Qair,rot = QO*_2_,*rot*). That leads to a constant overall flow rate (*Q_air_ + Q_O_*_2*,enr*_ *= const*) of 1.7 or 3.0 slpm regarding the experimental plan as indicated in Table 1. This principle has been explained in detail in another research [53]. In this case, different inlet air O_2_ enrichment levels were achieved by manipulating the oxygen valve open times:

(11) $$\tau_{O2,enr}=n_{O2,\%}\cdot \tau_{O2,period}/100$$

where *τ_O_*_2, period_ was 30 s, and *n_O_*_2_*,_%_*—oxygen valve percentage controlled by the PLC PID algorithm while maintaining the set DO level.

Enriching the inlet air with O_2_ and having one O_2_ gas analyzer at the exhaust gas line, inlet O_2_ concentration calculation is required. For this purpose, ‘inlet gas O_2_ calibration’ was made in a water environment to assess the O_2_ concentration in the inlet gas under different *n_O_*_2_,_%_ and when no oxygen consumption was present. Set calibration percentages (*n_O_*_2*_calibr*,%_) and the corresponding O_2_ concentrations in the output (*c_O_*_2*_calibr*,%_) were: *n_O_*_2*_calibr*,%_ = [0, 5, 10, 15, 20, 25, 30, 100], *c_O_*_2*_calibr*,%_ = [20.11, 26.25, 29.99, 33.84, 37.71, 41.62, 45.5, 100], respectively. Linear approximation of *c_O_*_2*_calibr*,%_ values between the measured calibration points were used for the whole *n_O_*_2*_calibr*,%_ interval with the step size of 1%.

The mathematical expressions of the necessary parameters for OUR and CPR calculation are described below. Pure oxygen flow rate at the moment of oxygen pulse (in L/min):

(12) $$Q_{O2,enr}=n_{O2,\%}\cdot Q_{O2,\;rot}/100$$

inlet air flow rate during an open air valve (L/min):

(13) $$Q_{air}=\left(1-n_{O2,\%}/100\right)\cdot Q_{air,\;rot}$$

correction factor taking into account the gas dilution by N_2_:

(14) $$CorF=\frac{C_{N2,air}\cdot Q_{air}}{\left(Q_{air}+Q_{O2,enr}\right)\cdot \left(100-C_{O2,air}-C_{CO2,air}\right)\cdot 100}$$

concentration of O_2_ in the inlet gas (vol. %):

(15) $$C_{O2,in}=\frac{\left(n_{O2,\%}-n_{O2\_calibr,\%}\left(i\right)\right)\cdot \left(C_{O2_{calibr},out}\left(i+1\right)-C_{O2_{calibr},out}\left(i\right)\right)\;}{n_{O2\_calibr,\%}\left(i+1\right)-n_{O2\_calibr,\%}\left(i\right)+C_{O2\_calibr,out}\left(i\right)}$$

total flow rate of O_2_ in the bioreactor (L/min):

(16) $$Q_{O2_{total},in}=\left(Q_{air}+Q_{O2,enr}\right)\cdot C_{O2,in}/100\;$$

oxygen transfer rate (g/kg/h):

(17) $$OTR\left(t\right)=\frac{\left(C_{O2,in}-\left(Q_{air}+Q_{O2,enr}\right)\cdot n_{O2,\%}\cdot CorF\right)\cdot M_{O2}}{100\cdot V_{m}\cdot W}$$

carbon dioxide production (evolution) rate (g/kg/h):

(18) $$CPR\left(t\right)=\frac{\left(-Q_{air}\cdot C_{CO2,air}/100+\left(Q_{air}+Q_{O2,enr}\right)\cdot n_{CO2,\%}\cdot CorF\right)\cdot M_{CO2}}{100\cdot V_{m}\cdot W}$$

### 2.7. Estimation of Biomass Concentration from OUR, CPR and BCR

In most aerobic cultivations, the relationship between the biomass concentration (*X*) and the OUR and CPR in a bioreactor can be modeled by means of Luedeking/Piret-type relationships [38,54]:

(19) $$OUR\left(t\right)=Y_{rXO}\cdot R_{X}\left(t\right)+Y_{mXO}\cdot X\left(t\right)$$

(20) $$CPR\left(t\right)=Y_{rXC}\cdot R_{X}\left(t\right)+Y_{mXC}\cdot X\left(t\right)$$

where *R_X_* is the biomass growth rate of the cellular system, g/kg/h; *X* is the biomass concentration, g/kg; and *Y_rXO_* [g(O_2_)/g(X)], *Y_rXC_* [g(CO_2_)/ g(X)] are yield parameters. *Y_mXO_* [g(O_2_)/g(X)/h] and *Y_mXC_* [g(CO_2_)/g(X)/h] are model parameters related to biomass maintenance as *Y_mXO_* quantifies the growth-independent part of the oxygen uptake rate and *Y_mXC_* quantifies the growth-independent part of the CPR. A similar equations can be formulated for the amonia or sodium hydroxide consumption rate during the cultivation [54] and for taking into account the influence of feed solution addition on the pH change [38]:

(21) $$BCR\left(t\right)=Y_{rXB1}\cdot R_{X}\left(t\right)+Y_{rXB2}\cdot \frac{F_{s}\left(t\right)}{W\left(t\right)}$$

where *Y_rXB_*_1_ [g(base)/g(X)] is the yield parameter, *Y_rXB_*_2_ [g(base)/kg(culture)] is a parameter related to the feeding (characterize the pH change due to substrate addition), and *F_s_* is the substrate feeding rate, kg/h; *W* is the culture mass (or volume in L if the culture broth density ≈1 kg/L), kg. The rate of *W(t)* change can be defined as:

(22) $$\frac{dW}{dt}=F_{s}+F_{b}+F_{af}-F_{smp}-F_{CO2}$$

where *F_b_* is the base addition rate, kg/h; *F_af_* is the anti-foam solution addition rate, kg/h; *F_smp_* is the sampling rate, kg/h; *F_CO_*_2_ is the carbon lost rate related to CO_2_ production/evolution, kg/h. As *W(t)* can be calculated online, the biomass balance in the reactor (Equation (23)) can be formulated by the ordinary differential Equations (24)–(26). Ordinary differential equations can be solved if the initial biomass *X*_0_ as well as the coefficients *Y_rXO_*, *Y_mXO_*, *Y_rXC_*, *Y_mXC_*, *Y_rXB_*_1_, and *Y_rXB_*_2_ are known. The six coefficients can be identified independently of the data records *W(t)*, OUR*(t)*, CPR*(t)*, BCR*(t)*, and *X(t)* previously measured in the process under consideration using standard nonlinear parameter optimization techniques [54]:

(23) $$\frac{dX}{dt}=R_{X}\left(t\right)$$

(24) $$\frac{dX}{dt}=\frac{OUR\left(t\right)-Y_{mXO}\cdot X\left(t\right)}{Y_{rXO}}$$

(25) $$\frac{dX}{dt}=\frac{CPR\left(t\right)-Y_{mXC}\cdot X\left(t\right)}{Y_{rXC}}$$

(26) $$\frac{dX}{dt}=\frac{BCR\left(t\right)-Y_{rXB2}\cdot \frac{F_{s}\left(t\right)}{W\left(t\right)}}{Y_{rXB1}}-\frac{F\left(t\right)}{W\left(t\right)}\cdot X\left(t\right)$$

Soft-sensor yield coefficients *Y_rXO_*, *Y_mXO_*, *Y_rXC_*, *Y_mXC_*, *Y_rXB_*_1_, and *Y_rXB_*_2_, used in the estimations, were determined using MATLAB’s *fminsearch* function minimizing RMSE between reference and soft-sensor output. Off-gas (O_2_/CO_2_ concentrations) and alkali consumption data available for all experiments presented in this research, was used to identify a set of individual soft-sensor parameter (*Y_rXO_*, *Y_mXO_*, *Y_rXC_*, *Y_mXC_*, *Y_rXB_*_1_, and *Y_rXB_*_2_) for correlation to DCW (results included in Appendix D). Off-gas analysis-based soft-sensors were fitted to the process data from both glycerol and methanol consumption phases. One set of alkali consumption-based soft-sensor parameters was identified for both substrate consumption phases, as during the initial process stage (corresponding to glycerol phase), alkali consumption dynamics had a weak correlation to biomass growth dynamics.

Obtained parameter sets were analyzed in context with the specific growth rate (Appendix D, Figure A10), as the soft-sensor yield and rate parameter dependency of specific growth rate was discussed in the Introduction section. However, strong correlation between the soft-sensor parameter sets from the experiments with similar growth rates (like for exps. 3c and 4c) or process conditions (like for exps. 4c and 5c) cannot be identified. The possible reason of this might be the posed inaccuracies due to OUR, CER or BCR calculation and varying cell metabolism in similarly propagating cultures under varying process conditions. As the scope of this research is to demonstrate model-free soft-sensor application possibilities under particular experimental conditions, techniques, like Kalman or Particle filtering are avoided. Soft-sensor yield parameter reconciliation procedures using first-principle (elemental balancing) constraints, leaving the soft-sensor techniques as simple as possible, are also avoided. Instead, a decision was made to include Mut^+^ strain cultivation experiments 3c, 4c, 5c and 6c, that have similar experimental conditions and consistent measurements available, in Dataset 3 for comparison reasons. The reference parameters from literature and the mean values of identified soft-sensor parameters for Dataset 3 experiments for both glycerol and methanol phases are presented in Table 3. The soft-sensor parameters obtained from Gamisans et al. research [43] (see Table 3), correspond to the specific biomass growth rate range of 0.035–0.150 1/h and 0.035–0.100 1/h for glycerol and methanol consumption phases, respectively.

**Figure 4:**
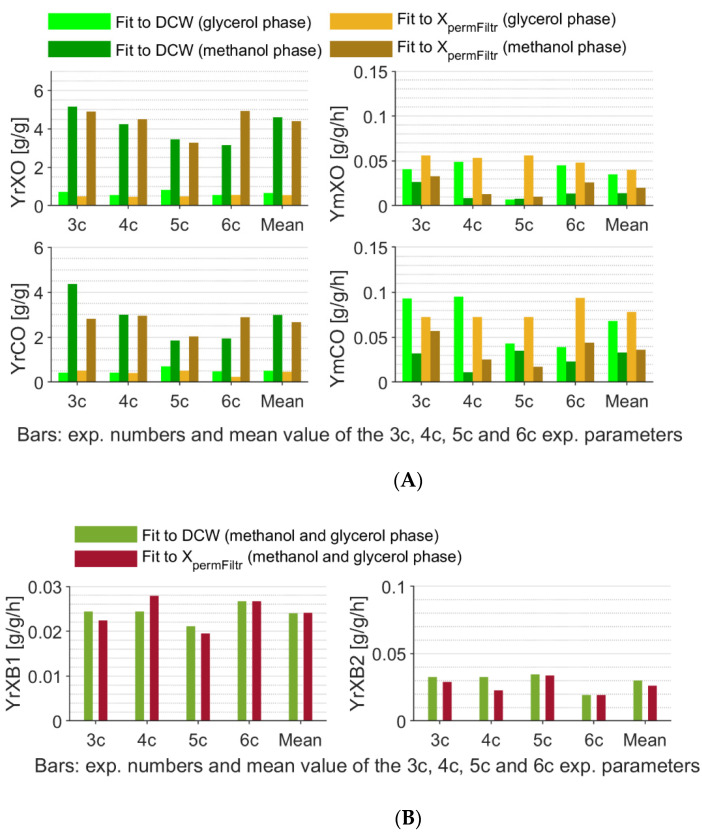
Identified individual and mean Dataset 3 soft-sensor parameters. (**A**) Off-gas analysis-based soft-sensor parameters. (**B**) Alkali consumption-based soft-sensor parameters.

Soft-sensor parameters for Dataset 3 experiments were also identified for permittivity-based reference biomass estimates, as this was found to be valuable for comparison reasons, assuming a more similar soft-sensor and permittivity measurement relation to the culture physiology in opposite to gravimetric DCW measurement. The identified soft-sensor parameters for both reference biomass measurements for Dataset 3 experiments and sole glycerol and methanol substrates are shown in Figure 4.

### 2.8. Estimation Quality Analysis

Estimation quality was analyzed by mean of normalized root mean square error attributed to the measurement range (NRMSE) and expressed in percent’s. Root mean square error expressed as:

(27) $$\mathrm{RMSE}=\;\sqrt{\frac{\sum_{i=1}^{n}{\left(X_{i}-{\hat{X}}_{i}\right)}^{2}}{n}}$$

and NRMSE:

(28) $$\mathrm{NRMSE}=\frac{\mathrm{RMSE}}{X_{max}-X_{min}}\cdot 100\%$$

where $X_{i}$ is the *i*th reference biomass measurement, ${\hat{X}}_{i}$ is the biomass estimate, $X_{min}$ and $X_{max}$ are the minimum and maximum values of reference $X_{i}$. NRMSE values, obtained in this research are calculated for the samples taken along the duration of the process (*t*_0_ − *t_end_*).

## 3. Results

Four datasets were used for the evaluation of the presented biomass concentration estimation method. Datasets consist of 13 cultivation experiments performed under various conditions, from which six were Mut^+^ (HBcAg production) and seven were Mut^S^ (HBsAg production) processes. The main results of the method implementation are presented further.

### 3.1. Biomass Concentration Determined Off-Line

**Figure A1:**
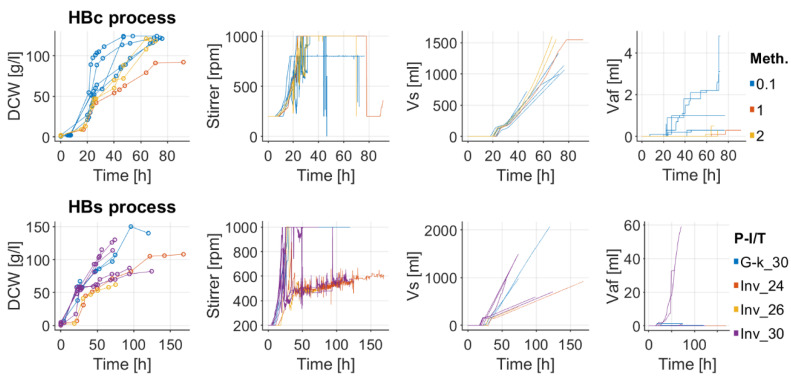
Some of the process variables regarding the process control strategy applied from Table 1. Legend description the same as for Figure A2.

**Figure A2:**
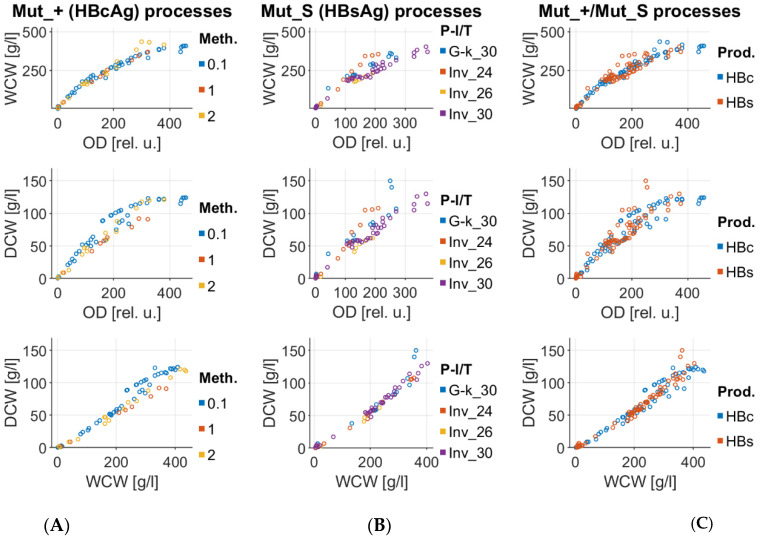
Correlation results of 3 different off-line biomass concentration detection methods: optical density (OD), wet cell weight (WCW) and dry cell weight (DCW) concentrations. The results contain the following notations: (**A**) HBcAg processes with different residual methanol level control in g/L (indicative concentrations according to Table 1); (**B**) HBsAg processes under different cultivation protocols and cultivation temperature (the legend indicates the abbreviation of the cultivation protocol and process temperature, respectively; information according to Table 1); and (**C**) comparison of panels A and B (HBc and HBs are short abbreviations of HBcAg and HBsAg, respectively).

Reliability of the off-line biomass optical density (OD), wet cell weight (WCW) and dry cell weight (DCW) concentration measurements can be evaluated on the basis of how these parameters correlate between each other. Information on the correlation between DCW, WCW and OD is important when recalculation from one to another measurement is necessary (for comparison reasons, yield calculations etc.). The results further described in detail are obtained from Dataset 1 and Dataset 2 off-line data included in Appendix A (Figure A2), where the method of correlation appears in a brief context of some of the varying process parameters indicated in Table 1 (some of the on-line parameters are available in Appendix A, Figure A1). The results indicates a more consistent and linear correlation of DCW~WCW compared to more disperse and less linear correlations of DCW~OD and WCW~OD (Figure A2, panel C). Two linear DCW~WCW correlation equations for the interval 0–200 WCW (R^2^ = 0.98, RMSE = 2.79 g/L):

(29) DCW=0.27·WCW

and for the interval 200–450 WCW/L (R^2^ = 0.88, RMSE 8.25 g/L)

(30) DCW=0.32·WCW−6.34

were identified.

Additionally, linear DCW~OD and WCW~OD approximation models were evaluated: DCW~OD within the OD interval 0–120 abs.u., DCW = 0.487*OD (R^2^ = 0.96, RMSE = 4.2 g/L) and WCW~OD within the OD interval 0–100 abs.u., WCW = 1.802*OD (R^2^ = 0.98, RMSE = 9.89 g/L).

Total (statistical and instrumental) measurement errors were evaluated from 1 experiment by repeating the analysis five times. Average OD measurement [1.2 2.9 36.4 119.0 219.2 251.0 322.4 362.2] values were calculated from samples taken along the process in different process stages for the whole density region, total respective OD measurement errors were ΔOD = [0.1 0.1 0.9 4.0 6.3 6.6 17.0 27.6]. DCW and WCW measurement total errors, compared to OD, deviated less along the whole process. Average ΔDCW and ΔWCW values were calculated as 0.98 DCW and 2.33 WCW, respectively. If the WCW measurement average error is recalculated to the dry biomass case (proportional coefficient ~0.3 as a mean from coefficients in Equations (27) and (28), the ΔWCW average measurement error of 0.7 DCW is about 30% lower than the measured ΔDCW. Despite this, the DCW concentration measurement was chosen to be correlated to the instrumental methods described further. This is due to the mathematical calculations used further, where DCW concentration is commonly used to represent specific yield and kinetic expressions.

### 3.2. Biomass Estimates from In-Situ Turbidity and Permittivity Sensors

**Figure 5:**
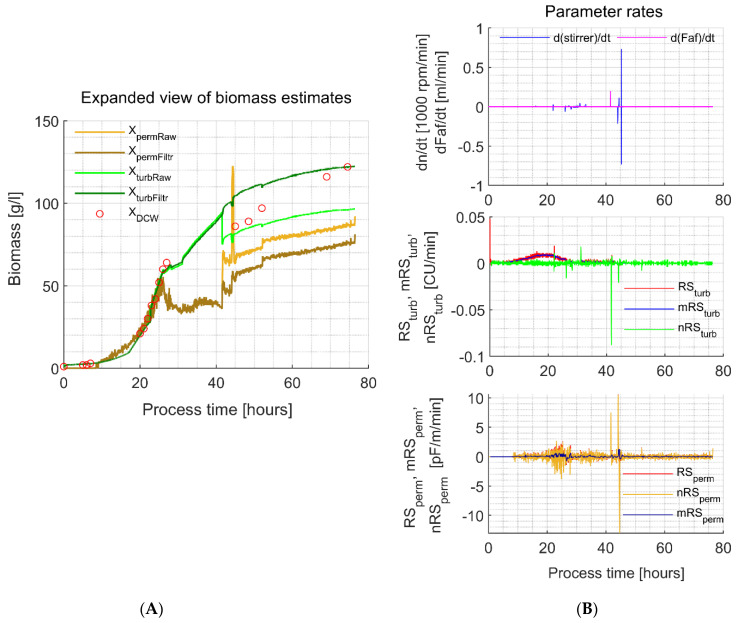
*In-situ* turbidity and permittivity sensor signal filtering parameter and result overview (experiment 6c). (**A**) Expanded view of the biomass estimates using raw and filtered turbidity and permittivity sensor signals. Plot on the top of panel B: changes in the stirrer rotational speed and antifoam solution addition rate. Plot in the middle of panel B: turbidity signal preprocessing parameters RS_turb_, mRS_turb_ and nRS_turb_ used in the filter. Plot in the bottom of panel B: permittivity signal preprocessing parameters RS_perm_, mRS_perm_ and nRS_perm_ used in the filter.

As it was discussed in the Introduction chapter, sudden stirrer rate changes and anti-foam agent addition may cause significant changes in the turbidity and permittivity signals and a respective shift in biomass estimates. Particular cross-sensitivity is presented in the example enclosed in Figure 5.

**Figure 6:**
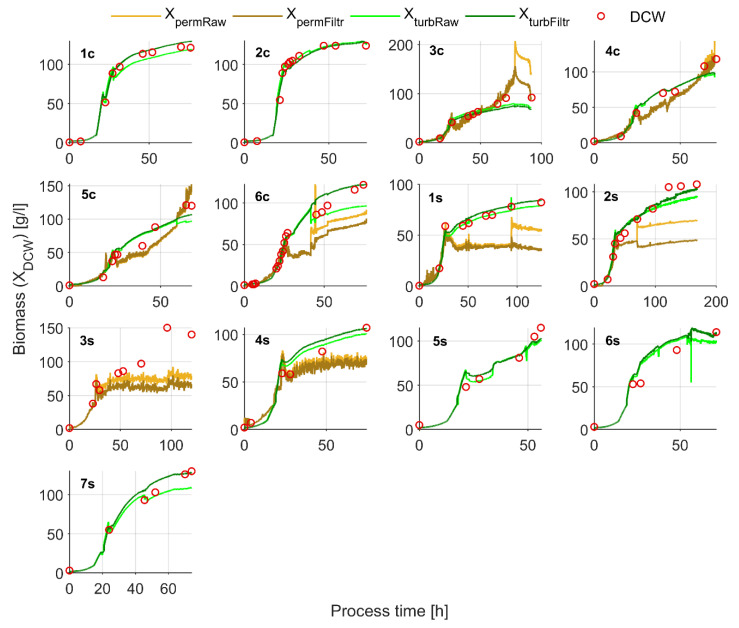
*In-situ* turbidity and permittivity sensor X_DCW_ estimation results.

**Figure A4:**
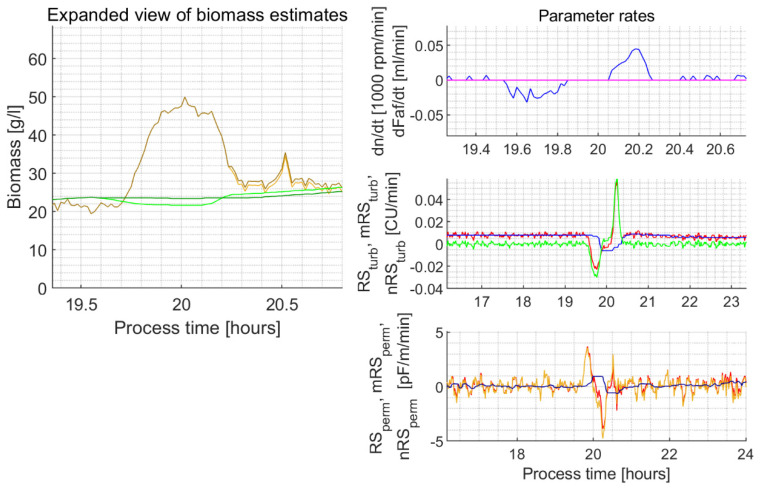
Experiment 5c; expanded view of the process 19.5–20.5 h. Line colors correspond to those identified in the Figures of the main text.

**Figure A5:**
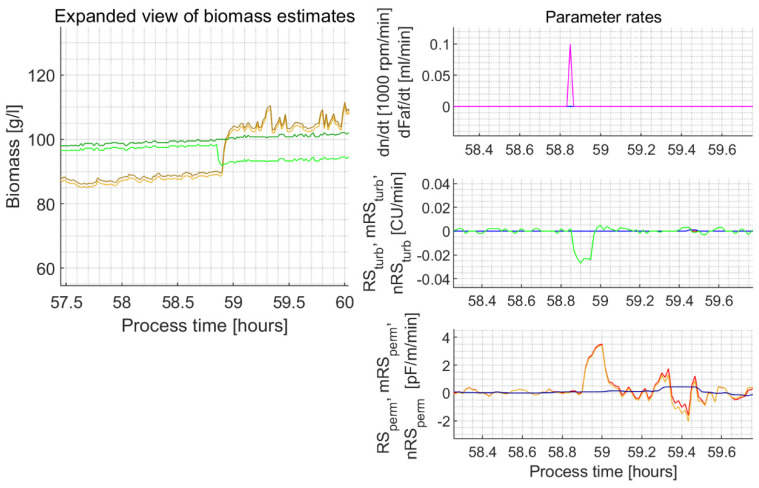
Experiment 5c; expanded view of the process 58–59.5 h. Line colors correspond to those identified in the Figures of the main text.

**Figure A6:**
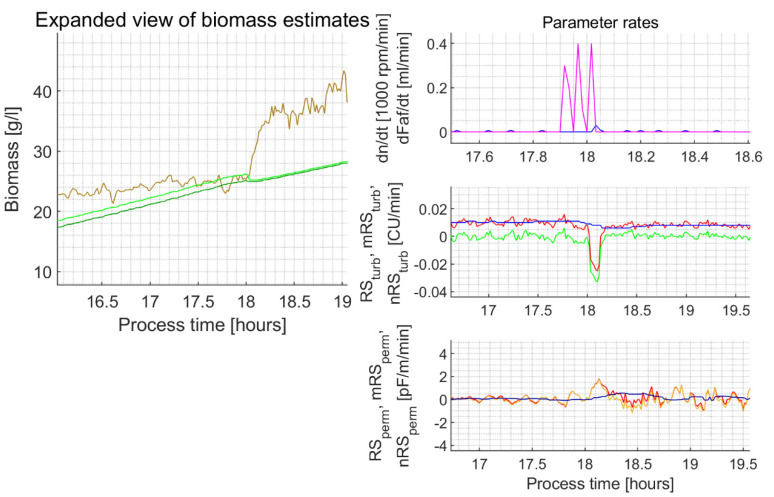
Experiment 4s; expanded view of the process 17–19 h. Line colors correspond to those identified in the Figures of the main text.

**Figure A7:**
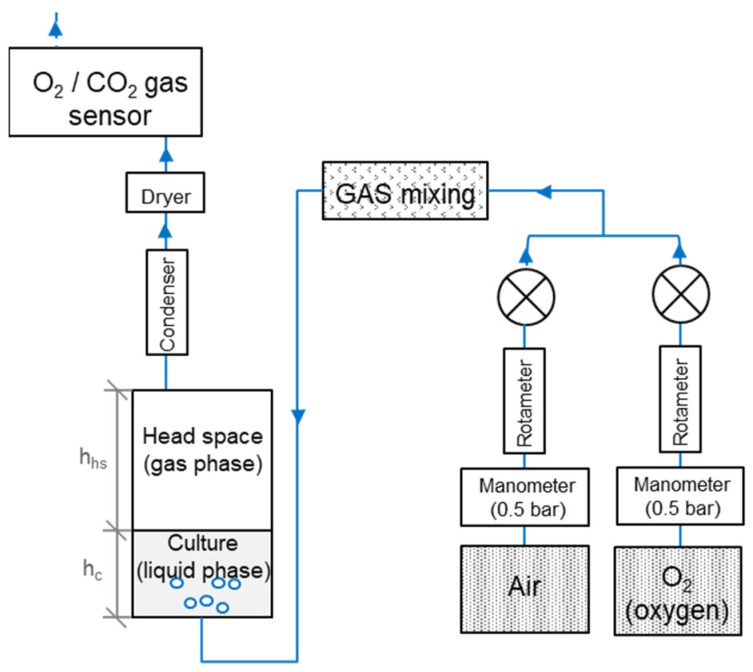
Flow chart of the used gas mixing system and exhaust gas line for gas analysis.

**Figure A10:**
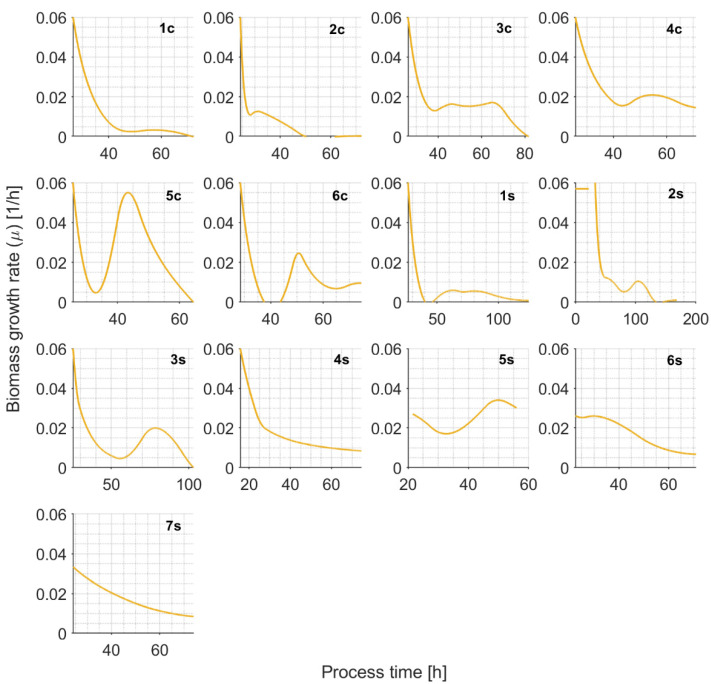
Specific growth rate calculated using two smoothing spline functions for close approximation of the growth trajectory between off-line DCW data points. The plots mainly show the specific growth rate from the methanol consumption phase, while appropriate specific growth rates for glycerol consumption phase were *μ_glyc_* = [0.18 0.17 0.09 0.10 0.14 0.14 0.21 0.06 0.13 0.31 0.11 0.13 0.12] 1/h (experiment order = [1c 2c 3c 4c 5c 6c 1s 2s 3s 4s 5s 6s 7s]).

For, example, well observable simultaneous negative turbidity and positive permittivity signal shifts (Figure 5, panel A) happen when the addition of an anti-foam agent takes place at around process 42 h (Figure 5, plot on the top of panel B). In the same example within process 44–45 h, a sudden stirrer rate decrease and an increase caused significant permittivity signal and minor turbidity signal shifts. As it can be seen from the supplementary data included in Appendix B (Table A1 and Figure A4, Figure A5 and Figure A6), a strong correlation of the shift actuator (stirrer rate or anti-foam addition rate change) and shift actuator signal height to the height of sensor signal shift or specific measurement (turbidity or permittivity), is not observable. This means, that the sensor signal should be analyzed directly for uncommon shifts that account for un-typical biomass growth/lysis. From the data presented in Figure 5 panel B, the nRS_turb_ and nRS_perm_ filtering criteria response to the shift actuators can be evaluated. Optimal (safe) filter parameters that are appropriate for accounting and filtering of the major turbidity and permittivity signal disturbances in Dataset 1/Dataset 2 and Dataset 3/Dataset 4 experiments respectively, were identified (Table 4) and the improvement for biomass concentration estimation quality, represented by *X_permFiltr_* and *X_turbFiltr_* results, can be visually observed in Figure 6.

As it can be observed from the results of experiment no. 6c (Figure 5, panel A, 42 h; or Figure 6, 42 h) and experiment no. 2s (Figure 6, ~75 h), the filter did not completely eliminate the sudden permittivity signal shifts. It would be possible to eliminate these sudden jump to a higher extent by narrowing the permittivity signal nRS_E,max_ and nRS_E,min_ bounds, although that would lead to signal over-filtration in some other explorative examples included in this research. Signal over-filtration is a result of the biomass increment/decrement-related sensor response ‘freezing’ in a no changing state (Figure 2, place in the algorithm where the actual sensor output is equalized to one-step-ahead measurement, *E_i,out_ = E_i-_*_1,*out*_). For example, some over-filtration can be observed for the turbidity signal at the end of experiment no. 6s and starting from the middle of experiment no. 7s. Over-filtration of the permittivity signal is not observable in such extent.

A total of six experiments (1c, 3, 5c, 6c, 1s and 4s), representing the average tendency of uncommon signal shifting frequency and range per experiment, were analyzed in detail, and the raw results are included in Appendix B. From 12 shift cases analyzed in detail, seven occurred due to the intensive change of the stirrer rotational speed, three resulted from the mixed stirrer/a-foam addition interaction, and two cases occurred purely because of anti-foam addition. In these analyzed examples, *X_turbRaw_* shifted by 10% on average, and the filter minimized this shift to 1.8%, but for *X_permRaw_*, it was 51.0% and 19.0%, respectively. From these results, an average filtering efficiency of the single shifts can be evaluated—for the turbidity measurement shifts, the error was reduced 5 times but for the permittivity measurement, this indicator was reduced twice.

A good *X_turbFiltr_* (for Dataset 1 and Dataset 2 experiments) and *X_permFiltr_* (for Dataset 3 and Dataset 4 experiments) estimation quality in the glycerol phase (average RMSE’s 5.0 and 2.7, respectively) and a notably lower estimation quality in the methanol phase (average RSME’s 12.3 and 28.5, respectively) was achieved. Normalized root mean square errors (NRSME) of 7% and 8% were calculated from Dataset 1/Dataset 2 and Dataset 3 *X_turbFiltr_* fit to DCW respectively. NRMSE of 11% was calculated from Dataset 3 *X_permFiltr_* fit to DCW. Calculated NRMSEs for *in-situ* and soft-sensors are compared in Table 5. Other results characterizing the soft-sensor performance, are presented in the next section.

Both methods showed a lower biomass estimation quality for the methanol consumption phase. Such behavior for *X_turb_* estimates can be explained by the application of the method based on limited optical characteristics under rather high cell density conditions. It should also be mentioned that, during the glycerol consumption phase, fewer off-line biomass samples were analyzed, therefore, having an impact on the estimation error calculated for this phase.

### 3.3. Biomass Estimation from OUR, CER and BCR Data

As it was shown in the soft-sensor development procedure in the Materials and Methods section, off-gas analysis-based soft-sensor yield (conversion) parameters for methylotrophic *P. pastoris* may depend on specific biomass growth rate. A strong correlation between the soft-sensor parameter sets from the experiments with similar growth rates (like for exps. 3c and 4c) or process conditions (like for exps. 4c and 5c) was not identified.

**Figure 7:**
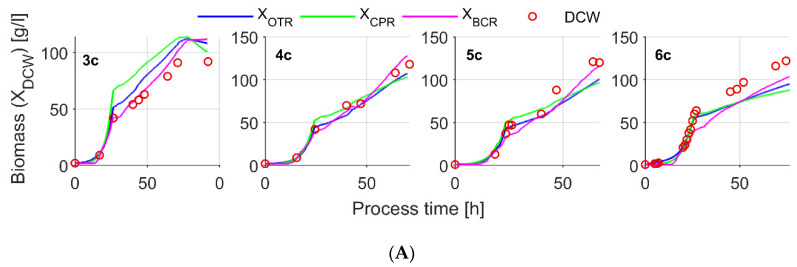

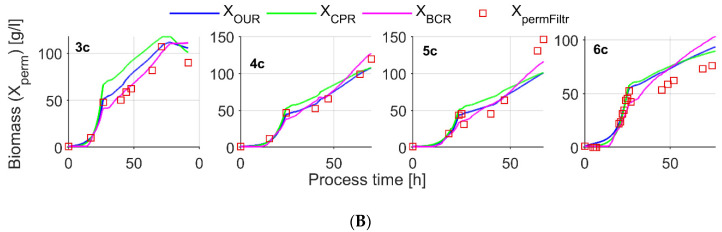
Soft-sensor biomass estimation results using mean values of identified yield coefficients presented in Table 3. Two different datasets fitted to: (**A**) off-line DCW (Dataset 3 and individual 3s experiments); and (**B**) sparse *X_permFiltr_* (Dataset 4 experiments).

Dataset 3 experiments with similar experimental conditions were selected for soft-sensor performance evaluation. Specific growth rates for Dataset 3 experiments were comparably similar, e.g., *μ_glyc_* = [0.09 0.10 0.14 0.14] 1/h for glycerol and *μ_meth_* = [0.015 0.015 0.030 0.010] 1/h for methanol consumption phase, respectively. The identified and reference yield and yield-rate parameter values are compared in Table 3. The identified model parameters were used for biomass estimation (results included in Figure 7).

**Figure A11:**
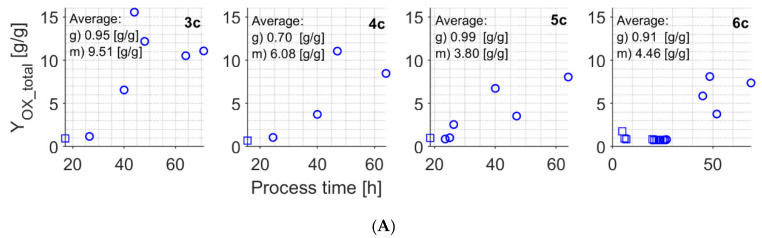

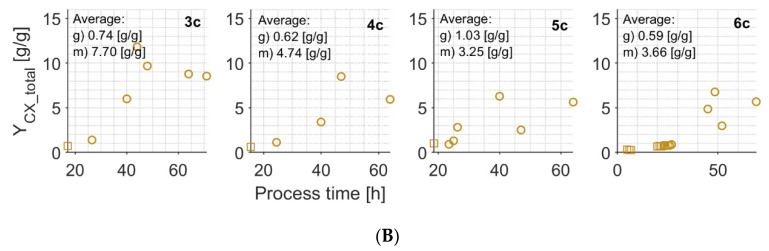
Total yield coefficients of O_2_ consumption (*YOX_total*) (panel A) and CO_2_ production (*YCX_total*) (panel B) for glycerol (squares) and methanol (circles) phases. In each subplot, average values from available measurement points for glycerol (g) and methanol (m) phases are indicated.

**Figure A12:**
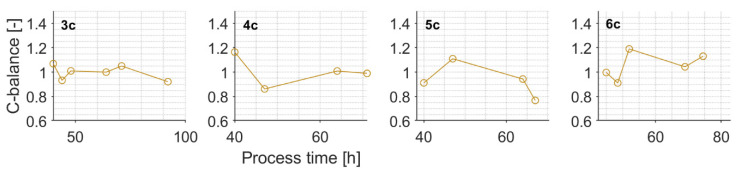
Carbon balance for the methanol consumption phase. Carbon balance calculated as a ratio of *carbon_accumulated_lost* / *carbon_supplied*. *carbon_accumulated_lost*, consisted of carbon utilized for biomass formation and carbon lost due to CO_2_ evolution; *carbon_supplied* consisted of carbon supplied due to methanol feed.

**Figure A13:**
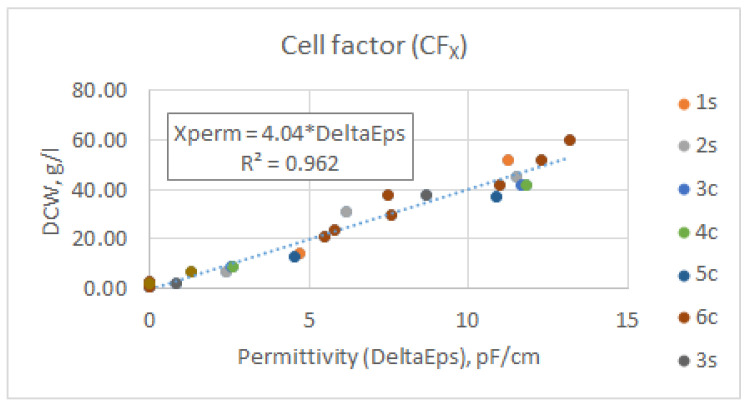
Permittivity signal correlation to DCW (fitted to Dataset 3 and 1s, 2s and 3s experiments).

As can be extracted from the available reference sources [39,43], O_2_ and CO_2_ yield parameters also include part of the consumed/produced O_2_/CO_2_ due to maintenance requirements. For comparison reasons, the integration of consumed O_2_ and produced CO_2_ per mass of biomass was done (data included in Appendix D, Figure A11). The mean yield coefficients *Y_OX_total_* and *Y_CX_total_*, representing the total yield ratio of consumed oxygen and produced carbon dioxide per mass of biomass, were in accordance or comparably close to the indicated reference values for those parameters identified for the methanol consumption phase. The *Y_CX_total_* parameter for glycerol consumption phase is also close to the reference sources, however it partly declines, indicating an unclosing C-balance. As it appears from the carbon mass balance for the methanol consumption phase (Figure A12 enclosed in the Appendix D), for the majority of reference DCW measurements, a C imbalance is lower than 10%, indicating for a closing C elemental balance that was also proposed elsewhere [39]. The findings above indicate a comparably accurate CER calculation. At the same time, a comparably higher OUR-based yield coefficient deviation from reference sources indicate for inaccuracies in OUR calculations. Such inaccuracies may occur due to O_2_ off-gas measurement calibration shifts and/or an insufficiently accurate method utilized for oxygen concentration calculation in oxygen enriched inlet air.

With the identified set of Dataset 3 mean parameters, DCW estimation error (NRMSE) for *X_OUR_*, *X_CPR_* and *X_BCR_* was 10%, 13% and 8%, respectively. The soft-sensor parameters for fitting to the reference permittivity (*X_permFiltr_*) measurement, identified for the same dataset, did not lead to an improved estimation quality for any of the observed methods. The estimation quality between the used methods is compared in the Table 5.

## 4. Discussion

Various biomass estimation methods were applied in up to 135 DCW high cell density *P. pastoris* cultivations under varying process conditions. A high number of cultivations (13 exp.) were analyzed. This forms an extensive overview of the method reproducibility and applicability under particular or similar experimental conditions. As the glycerol or methanol consumption leads to different cell physiological behavior, separate soft-sensor parameters were fit for each of the substrate consumption phases. Below, the major findings are summarized, and a discussion on the result interpretation is extended.

### 4.1. Off-Line Biomass Detection Methods

For cell densities up to 60 DCW, the photometric OD measurements suitably correlate to the DCW (RMSE 4.2 g/L). For higher DCW densities, the OD measurement fails to sufficiently represent the biomass dynamics. The above-mentioned is true because of the cross-sensitivity of the well-known UV/NIR method to culture constituents, such as cell debris, by-products or other matters added to the bioreactor. The aforementioned is illustrated in an obvious way elsewhere [27], where the same *in-situ* turbidity signal at different process stages corresponds to about 2 times different DCW levels. Statistical and instrumental errors of ±0.98 DCW and ±2.33 WCW obtained for DCW and WCW measurements respectively, indicate a suitable accuracy for reference biomass concentration measurement methods.

### 4.2. DCW Estimation with the In-Situ Turbidity Sensor

Biomass densities of 50 DCW were achieved up until the start of the methanol consumption (induction) phase. For biomass densities up to 50 DCW, the *in-situ* turbidity method performed well for DCW estimation (RMSE 5.0 g/L). For the higher biomass densities of 50–135 g/L, an estimation accuracy about two times lower was observed (RMSE 12.3 g/L). Few research results are available, where turbidity probe applications are demonstrated at such high cell densities (≥90 DCW). In the research conducted by Goldfeld and co-authors [30], at-line NIR turbidity measurement was used for high cell density *P. pastoris* biomass monitoring. A similar correlation quality up to about 90 DCW (300 WCW) can be observed for the turbidity related biomass estimation method [30]. However, for the 90–165 DCW (300–550 WCW) interval, the average deviation between off-line (WCW) and on-line measurements fit either poorly or even not at all [30]. From the available measurement data [30], a rough estimate of at least 1.5 times better X_turbFiltr_~DCW fit for the 90–130 DCW range, comprehensively examined in the current research, can be evaluated for the current contribution. Another research is available, where successful *S. cerevisiae* monitoring for up to 90 DCW [23] is presented. However, in this example, a long-lasting (45 days) continuous membrane filtrated culture under constant mixing and aeration conditions was investigated. As one of the discussed reference examples had a lower turbidity-based biomass estimate correlation quality to DCW [30], but the other one [23] lacked an investigation in the biomass density region above 90 DCW, the particular contribution can be addressed as one of the rare examples, where the turbidity-based biomass estimation results above 90 DCW are demonstrated with comparably higher accuracy. Moreover, a new region of high cell densities for the *in-situ* turbidity technique is investigated. Turbidity measurement demonstrated one of the highest accuracies of 8% (NRMSE) in comparison to other investigated methods.

### 4.3. In-Situ Permittivity (X_permFiltr_) Based Biomass Estimates

For the glycerol consumption phase (<25–30 h), permittivity-based biomass estimates fit well to DCW. Starting from the very beginning of the methanol consumption phase, within 1–2 h, X_permFiltr_ declined in all cultivations by about 10–20 g/L. This change in the culture dielectric properties under new conditions is a characteristic behavior as adaptation to another substrate and start of recombinant protein synthesis occurs [57]. The X_permFiltr_ behavior within the methanol consumption phase differed between Mut^+^ and Mut^S^ strains. In the majority of Mut^+^ processes (3c, 4c and 5c), X_permFiltr_ closely followed the DCW dynamics. At the same time, in the Mut^S^ processes, X_permFiltr_ remained at the same level (1s and 3s) or increased slowly (2s and 4s). Similar permittivity-based biomass estimates and DCW dynamics are observable also in the research conducted by Goldfeld et al. [30].

The reason for such X_permFiltr_ and DCW shifts in the comparably similar processes (like for the Dataset 3 experiments) can be caused by the varying permittivity of cell population caused by changes in the cell size and/or intracellular conductivity [57]. If one assumes that the intracellular conductivity remains unchanged for the morphologically different cell clusters, the varying dynamics of the cell population size could lead to differences between X_permFiltr_ and DCW. Vanz et al. [49] studied the morphological changes of *P. pastoris* under high yield HBsAg production. From the presented data, one can extract that, during the induction phase (120 h) for 40% of the cell population, the diameter increased by about 50% leading to about 3-fold increased cell volume for this population. Particular changes at the end lead to the cell volume increase of the whole system by about 20%. At the same time, the number of apoptotic cells in the induction phase increased by 10%. Overall, that would lead to a viable cell volume increase by 10%. Under circumstances introduced earlier, the permittivity-based biomass estimate should, accordingly, increase by 10%. Raschmanová et al. [31] extensively studied the morphological changes of several *P. pastoris* strains under different growth rates, synthesizing extracellular proteins. The results indicate the extended range for the apoptotic (impaired viability) cells to form 10 to 30% of the population depending on the strain used and the specific biomass growth rate. Moreover, the populations of larger cells appeared to grow under medium biomass growth rates (*μ_methanol_* = 0.16 1/h), and with a trend to decrease at low (*μ_methanol_* = 0.08 1/h) and high (*μ_methanol_* = 0.32 1/h) biomass growth rates.

The findings above show that the *P. pastoris* culture morphology and viability significantly change over the methanol induction phase. Moreover, the dynamics of these changes vary under different biomass-specific growth rates (excess methanol levels), influencing also the permittivity-based biomass estimate correlation to DCW. This could be the case of the varied X_permFiltr_ and DCW correlation for Mut^+^ cultivations presented here, as the protein yield and accumulation dynamics were similar in these processes (yet unpublished results). Despite the varied cultivation conditions in Mut^S^ cultivations, a significant decline in X_permFiltr_ against DCW was noted. Due to the poor HBsAg biosynthesis, the obtainment of purified product was possible from only one experiment (yet unpublished results). This leads to the conclusion that in the Mut^S^ processes, less viable, low-size and low-productivity cell populations dominated in comparison to Mut^+^ processes.

A number of permittivity measurements showed an average DCW fit quality of 11% (NRMSE) for Dataset 3 using fixed parameter sets for glycerol and methanol consumption phases. Above-described permittivity measurement performance lead to the conclusion, that there is some interpretation gap, regarding permittivity correlation to methylotrophic *P. pastoris* DCW biomass during induction phase. Therefore, for the in-depth investigation of permittivity/DCW correlation, analysis of the cell morphology change would be necessary.

### 4.4. Soft-Sensor-Based Biomass Estimates

Evaluated off-gas and alkali consumption-based soft-sensor estimation accuracies for Dataset 3 experiments are 10%, 13% and 8% for oxygen-uptake-based, carbon-production-based and alkali-consumption-based biomass estimators, respectively. As the scope of this research was to demonstrate a model-free soft-sensor development that is as simple as possible, the obtained results still could be improved by means of the methods discussed in the introduction. Taking into account the differences between some of the identified and reference yield parameters for oxygen consumption and, in a lesser extent, for carbon dioxide production, one should state that the possible reasons for this could be related to the imprecisions in O_2_/CO_2_ off-gas sensor calibration, airflow adjustment etc. For oxygen consumption-based yield parameters, these differences may also occur due to an insufficiently accurate method utilized for oxygen concentration calculation in oxygen-enriched inlet air.

The alkali consumption-based estimator shows similar performance to turbidity measurement. Knowing that the turbidity measurement better performs in the first part of the process (<50 DCW), but alkali-based biomass estimator indicates better results starting from the end of the glycerol batch phase (>25 DCW), a combination of both measurements would lead to a superior, reliable and non-complex biomass estimation procedure.

### 4.5. In-Situ Turbidity and Permittivity Signal Filter

The *in-situ* sensor signal filtering method lead to about 5-fold and 2-fold minimized biomass estimate drifts for turbidity- and permittivity-based biomass estimates, respectively. As the method was verified in a sufficiently high number of experiments (for turbidity 12 exps. and for permittivity 8 exps.), it is applicable in process on-line monitoring. Considering filtering performance, e.g., some of the shifts may be filtered only partly or some overfiltration is possible, the method is applicable for decision making in bioprocess control. Some examples of unfiltered permittivity signal peaks, analyzed in detail, are added to the supplementary material (Appendix B, Figure A4, Figure A5 and Figure A6). From this data, one can observe that unfiltrated shift nRS_E_ does not exceed preset reference nRS_E,max_ and nRS_E,min_ bounds, but the nRS_E_ peak has a wider area compared to neighbor peaks not related to the signal uncommon shift. Therefore, identifying uncommon shift nRS_E_ peak threshold area and selecting it as a filtering criterium, might lead to improved filtering accuracy.

### 4.6. Concluding Remarks

Despite the inert implementation of the process analytical tools for decision-making in biopharma’s process control, the future cybernetical-physical systems (i.e., interconnected systems of physical machines that are controlled by soft-sensor and algorithms) are likely to become autonomous units, being able to function without manual interventions, and delivering quality by design [58]. For that purpose, reliable and well interpretable real-time biomass sensor data acquisition will be of major importance. Due to this reason, the contribution authors expect to enhance the development of biomass monitoring.

## Figures and Tables

**Table 1:** Experiment overview.

Exps.^1^	Cultivation Protocol	Parameter Settings			Variable Control Range in the Process	
		Temperature (°C)	Aeration (*Q_air_*) (slpm)	O_2_ Enrich-ment	Residual Methanol ^2^ (g/L)	Dissolved Oxygen (DO) ^3^ (%)
1c	Inv., Mut^+^	30	1.7	yes	0.05–0.1	35–40
2c	Inv., Mut^+^	30	1.7	yes	0.01–0.05	25–40
3c	Inv., Mut^+^	30	3.0	yes	0.5–1.5	25–30
4c	Inv., Mut^+^	30	3.0	yes	1.5–2	25–30
5c	Inv., Mut^+^	30	3.0	yes	1.5–2	25–30
6c	Inv., Mut^+^	30	3.0	yes	0.02	25–30
1s	Inv., Mut^S^	30	3.0	yes	0.01	20–40
2s	Inv., Mut^S^	24	3.0	yes	0.01	20–40
3s	Gurramkonda	30	3.0	no	5–7	3–5
4s	Gurramkonda	30	3.0	no	1–3	15–25
5s	Inv., Mut^S^	30	3.0	yes	0.5–2.0	25–30
6s	Inv., Mut^S^	30	3.0	no	4– 5	1–2
7s	Inv., Mut^S^	30	3.0	no	1.5–2.5	1–2; 20–35

^1^ The letters indicate specific strain cultivated—‘c’ HBcAg obtainment processes and ‘s’ HBsAg obtainment processes. ^2^ Residual methanol—an indicative methanol concentration range in the methanol consumption (induction) phase. On-line and off-line methanol analysis is available in Appendix A, Figure A3. ^3^ Some of the online process parameters are indicated in Appendix A, Figure A1.

**Table 2:** Identified *in-situ* turbidity sensor biomass estimation approximation models and their parameters for two aeration regimes.

Datasets of Experiments	Aeration (slpm)	Fit Interval	Model Name	Model Parameters
Dataset 1: 3c, 4c, 5c, 6c, 1s, 2s, 4s, 5s, 6s, 7s	3.0	Whole region	*Exponential 1* (Equation (7))	a = 1.547, b = 2.85
Dataset 2: 1c, 2c	1.7	*E_turb_* ≤ 1.40	*Exponential 2* (Equation (8))	a = 3939, b = 4.8, c = −3938, d = 4.8
		*E_turb_* ≤ 0.72	*Linear* (Equation (9))	a = 10.76, b = 2.176
		*E_turb_* > 0.72	*Linear* (Equation (9))	a = 542.3, b = −109

**Table 3:** Soft-sensor yield coefficients attributed to DCW.

Symbol	Unit	Value			
		Glycerol (Identified ^1^)	Glycerol (Reference)	Methanol (Identified ^1^)	Methanol (Reference)
*Y_rOX_*	g/g	0.66	—	4.60	—
*Y_mOX_*	g/g/h	0.035	0.026 ^3^	0.014	0.003 ^3^ 0.020 [17] 0.024–0.045 ^4^
*Y_OX_total_* ^2^	g/g	0.70–0.99	1.42–1.79 [43]	3.80–6.08	5.69–6.51 [43] ~5.5 [39]
*Y_rCX_*	g/g	0.51	—	2.99	—
*Y_mCX_*	g/g/h	0.068	0.071 [55] 0.166 ^3^	0.033	0.282 [55] 0.029 ^3^
*Y_CX_total_* ^2^	g/g	0.59–1.03	1.17–1.59 [43]	3.25–4.74	4.01–4.78 [43] ~3.5 [39]
*Y_rBX_*_1_	g/g/h	0.024	—	0.024	—
*Y_rBX_*_2_	g/g/h	0.030	—	0.030	—

^1^ Mean values of identified Dataset 3 parameters. *Y_OX_total_* and *Y_CX_total_* average parameter value intervals obtained from exps. 4c, 5c and 6c (data included in Appendix D, Figure A11); average parameters from 3c exp. were excluded, as they significantly differ from the reference for the methanol phase. ^2^ Total yield also includes consumed oxygen or produced CO_2_ due to culture maintenance requirement. ^3^ Parameters calculated from *Y_mOX_ = m_ATP_ ∙ Y_O_*_2*/ATP*_ *∙ M_O_*_2_ and *Y_mOX_ = m_ATP_ ∙ Y_O_*_2*/ATP*_ *∙ M_O_*_2_ equations for O_2_ uptake and CO_2_ production cases respectively (m_ATP_ 2.51 mmol(ATP)/g(X)/h and 0.44 mmol(ATP)/g(X)/h for glycerol and methanol consumption phases respectively taken from Gamisans et al. research [43]); stoichiometric yield coefficients *Y_O_*_2*/ATP*_ = 0.5/1.53 = 0.33 g(O_2_)/g(ATP) and *Y_CO_*_2*/ATP*_ = 3/2 = 1.5 g(CO_2_)/g(ATP) for glycerol and *Y_O_*_2*/ATP*_ = 0.5/2.01 = 0.25 g(O_2_)/g(ATP) and *Y_CO_*_2*/ATP*_ =3/2 = 1.5 g(CO_2_)/g(ATP) for methanol consumption cases respectively adapted from Niu et al. research [55]. ^4^ Calculated using the available range from the reviewed maintenance rate coefficients 0.016–0.030 g(S)/g(X)/h [56] and *Y_O_*_2*/S*_ = 1.5 g(O_2_)/g(S) [17].

**Table 4:** Upper and lower normalized rate sum (nRS_E,max_/nRS_E,min_) bounds used for on-line turbidity and permittivity signal (*E*) filtering.

	nRS_E_,_max_/nRS_E_,_min_	*τ_sum_*	*τ_med_*
For the turbidity signal	0.01/−0.01 CU/min	5 min	30 min
For the permittivity signal	2.5/−2.5 pF/m/min	5 min	30 min

**Table 5:** Normalized root mean square error (NRMSE) values fitted to DCW and *X_permFiltr_* for different datasets.

	*X_turbFiltr_*	*X_permFiltr_*	*X_OUR_*	*X_CPR_*	*X_BCR_*
	Average NRMSE (%)				
Fit to DCW for Dataset 1 and Dataset 2	7	—	—	—	—
Fit to DCW for Dataset 3	8	11	10	13	8
Fit to *X_permFiltr_* for Dataset 3	—	—	11	14	10

**Figure A3:**
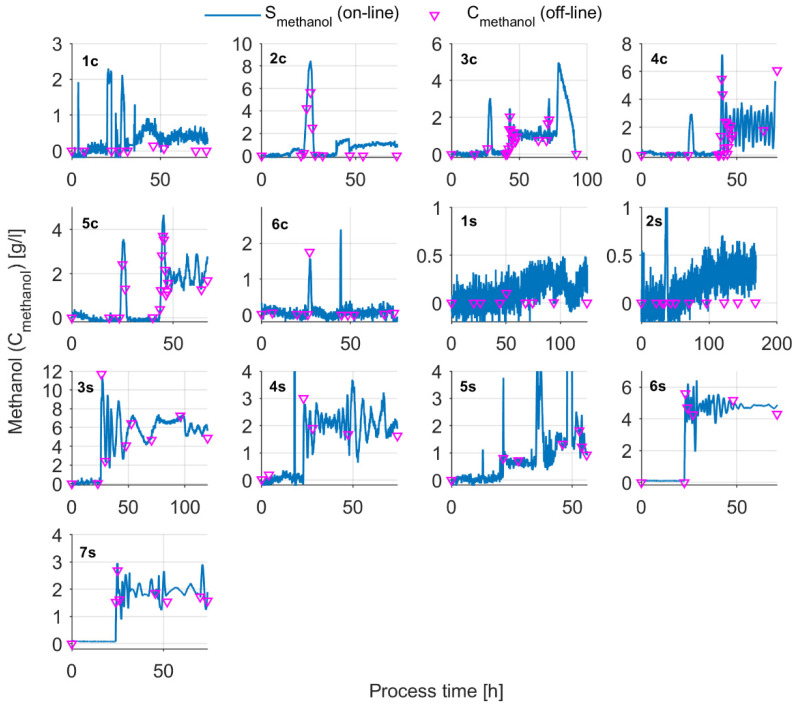
On-line and off-line methanol analysis. Online methanol in the processes 6s and 7 s, monitored with *in-situ* methanol probe (Raven); the rest of the on-line methanol data are from methanol vapor off-gas analysis (BCP-EtOH, Bluesens).

**Figure A8:**
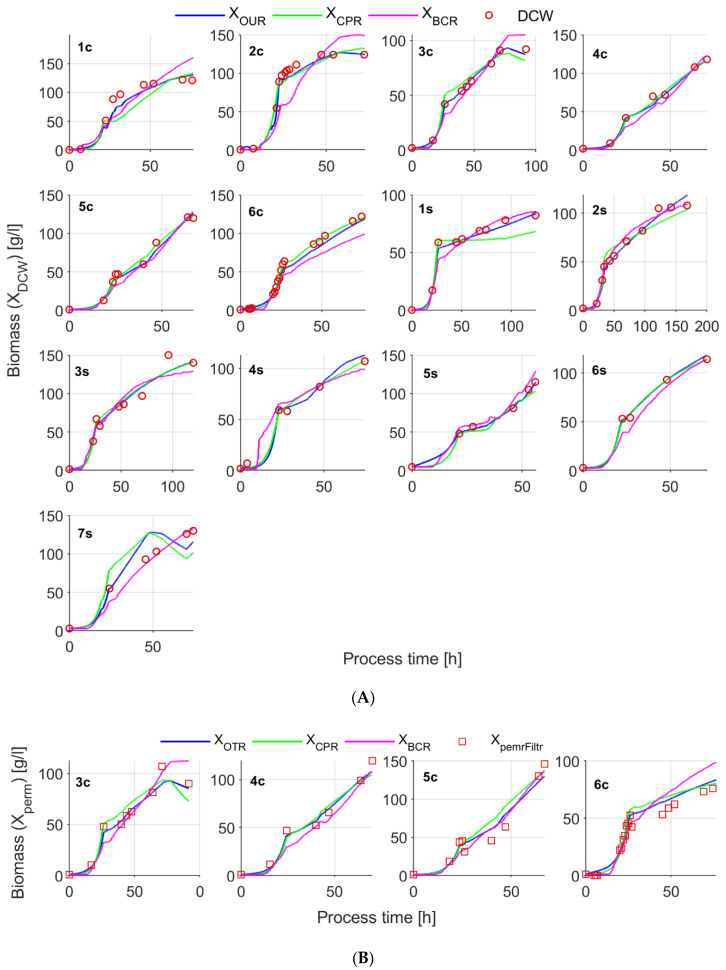
Soft-sensor biomass estimation results, using individually identified yield coefficients. Two different datasets fitted to: (**A**) off-line DCW (Dataset 1/Dataset 2 and individual 3s experiments) using coefficients presented in Figure A9; and (**B**) sparse *X_permFiltr_* (Dataset 4 experiments) using coefficients presented in Figure 4.

**Figure A9:**
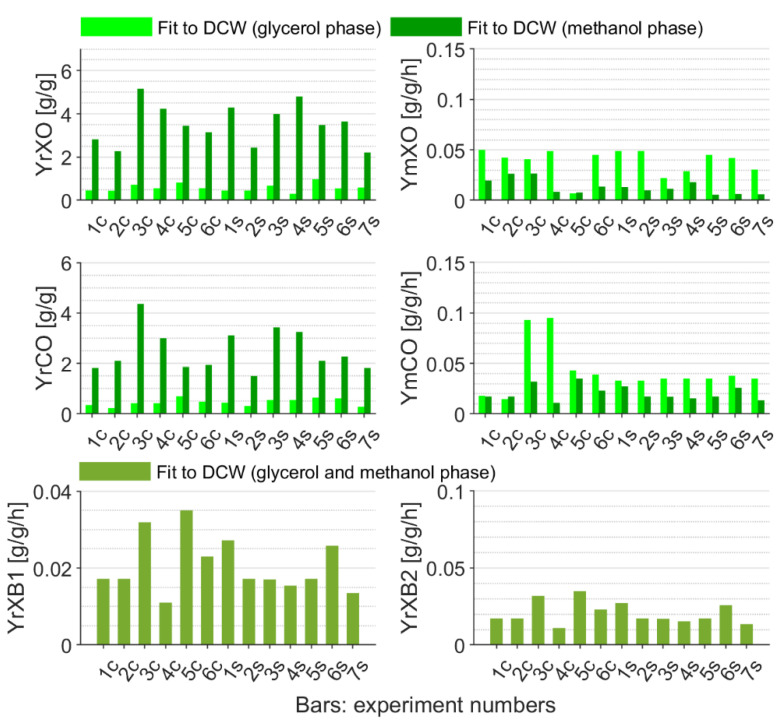
Identified individual Dataset 1, Dataset 2 and 3s experiment soft-sensor parameters.

## Data Availability

Data sharing is not applicable to this article.

## List of Symbols

CF	Cell factor [g/L/pF/cm]
HBcAg	Hepatitis B core-antigen
HBsAg	Hepatitis B surface-antigen
DO	Dissolved oxygen
OD	Optical density [rel. u. (relative units)]
DCW	Dry cell weight concentration [g/L]
WCW	Wet cell weight concentration [g/L]
GMP	Good Manufacturing Practice
PID	Proportional, Integral and Derivative control parameters
PLC	Process Logical Controller
SCADA	Supervisory Control and Data Acquisition
*c*	Concentration
O_2_	Oxygen
CO_2_	Carbon dioxide
N_2_	Nitrogen
*μ*	Biomass growth rate [1/h]
*Fs*	Feeding rate of substrate solution [kg/h]
*F*_*CO*2_	Carbon loss via off-gass [kg/h]
*F*_b_	Alkali addition rate [kg/h]
*F*_e_	Evaporation rate [kg/h]
*F_smp_*	Sampling rate [kg/h])
*E*	Raw sensor signal (output) [units depend on the sensor]
*E_perm_*	Permittivity sensor signal (output) (DeltaEps) [pF/cm]
*E_turb_*	Turbidity sensor signal (output) [CU (concentration units)]
R	Rate
RS	Rate sum
R_X_	Biomass formation rate [kg(X)/kg(Culture)/h]
*Q_air_*	Air flow rate [slpm (standard liters per minute)]
*Q*_*O*2,*enr*_	Oxygen flow rate [slpm (standard liters per minute)]
*t*	Process time [h]
τ	Period [s, min, h]
W	Culture mass [kg]
x	Total biomass weight [g, kg]
X	Biomass concentration [g/L, kg/L]
Y	Yield parameter [kg/kg]
**Indices**	
ATP	Adenosine triphosphate
*i*	Item number *i*
*t*	Time point *t*
O, O_2_	Oxygen
C, CO_2_	Carbon dioxide
*E*	Raw sensor signal (output)
B	Base or alkali
*X*	Biomass
DCW	Dry cell weight concentration
WCW	Wet cell weight concentration
af	Antifoam
*m*	Maintenance
*S*	Substrate
enr	Enrichment
Filtr	Filtrated signal
%	Percent
calibr	Calibration
pH	Acidity measure
feed	Feed solution
turb	Turbidity
perm	Permittivity
glyc	Glycerol
meth	Methanol
end	End measurement
0	Initial measurement

## Appendix B

**Table A1:** *In-situ* turbidity and permittivity sensor signal filtering quality parameter evaluation overview for a part of the presented experiments.

Column. no. (→)	1	2	3	4	5	6	7	8	9
Exps.	Time (h)	Max. dn/dt (rpm)	Max. d(a-foam)/dt mL/min	Max. RSn for *E_turb_* (min)	Biomass Estimates *X_turbRaw_*_0_ *X_turbRaw_*_1_ (Diff. in %) (g(Dry Cell Weight)/L)	Biomass Estimates *X_turbFiltr_*_0_ *X_turbFiltr_*_1_ (Diff. in %) (g(Dry Cell Weight)/L)	Max. RSn for *E_perm_* (min)	Biomass Estimates *X_permRaw_*_0_*X_permRaw_*_1_ (Diff. in %) (g(Dry Cell Weight)/L)	Biomass Estimates *X_permFiltr_*_0_*X_permFiltr_*_1_ (Diff. in %) (g(Dry Cell Weight)/L)
1c	21.3	−97	0	−0.069	65.08 53.6 (17.6%)	62.3 59.1 (5.1%)	**–**	**–**	**–**
	23.05	139	0	+0.069	51.8 63.6 (22.8%)	58.658.6(0%)	**–**	**–**	**–**
	27.53	70	0.9 (27.47 h)	−0.099	97.2 79.7 (18%)	91.3 91.3 (0%)	**–**	**–**	**–**
2c	NA	NA	NA	NA	Good filtering quality	NA	NA	NA	NA
3c	22.7	36	0	+0.014	25.7 26.4 (2.7%)	25.7 25.7 (0%)	+0.67	NA	NA
	78.05	−131	0.2 (77.38 h)	+0.014	78.7 78.0 (0.9%)	73.8 74.0 (0.3%)	+12.3	140.9 206.1 (46.3%)	155.2 152.8 (1.5%)
4c	Similar to 3c experiment								
5c	19.73–20.30	NA	NA	NA	NA	NA	In the explorative experiments, the unusually lasting decrease and then the lasting increase of the stirrer rotation speed lead to a permittivity signal peak unfiltered by the proposed filter algorithm and filter parameters (Figure A3)		
	58.83	0	0.1	−0.023	99.5 91.8 (7.7%)	100.0 100.0 (0%)	+3.5	90.1 101.1 (12.2%)	90.1 101.1 (12.2%)
6c	36.8	0	0.2	−0.088	94.5 75.5 (20.1%)	93.3 96.7 (3.6%)	+7.5	41.6 69.2 (66.3%)	41.6 50.9 (22.4%)
	44.07	−217	0	−0.019	81.7 76.7 (6.1%)	100.8 98.2 (2.6%)	+10.1	66.21 121.9 (84.1%)	46.9 56.6 (20.7%)
	44.50	114	0	0.0005	76.1 80.8 (6.2%)	98.0 103.7 (5.8%)	−13.1	118.8 64.8 (45.5%)	57.2 53.9 (5.8%)
1s	94.03	62	0	−0.039	87.4 78.0 (10.8%)	80.6 80.6 (0%)	3.99	37.7 67.9 (80.1%)	37.7 45.2 (19.9%)
4s	18.03	30	17.9–18.02 h tree ~0.35 mL impulses of a-foam	−0.035	26.2 25.3 (3.4%)	25.1 25.0 (0.4%)	1.78 (max. reached in ~6 min)	26.06 34.3 (31.6%)	26.06 34.3 (31.6%)
	22.25	26	0	−0.030	71.2 66.1 (7.2%)	70.0 68.8 (1.7%)	+4.40	42.7 60.5 (41.7%)	42.7 60.5 (41.7%)
**Mean difference in %**					**10.3%**	**1.6%**	—	**51.0%**	**19.5%**

NA: not analyzed. -: no measurement. Mean difference in %: mean of differences indicated in parentheses. Description of the columns of Table A1: (1) Time when a significant sensor signal change starts; (2) Stirrer maximum change rate reached in approximately 2 to 3 min starting from the time indicated in column 1; (3) Maximum addition rate of the anti-foam agent reached in approximately 2 to 3 min starting from the time indicated in column 1 or reached in the time indicated in parentheses (from the right of the parameter); (4) Maximum RSn of the turbidity signal reached in approximately 2 to 3 min starting from the time indicated in column 1; (5) Influence of the raw sensor signal shift of DCW biomass estimate, where: X_turbRaw0_ biomass estimate from the raw turbidity sensor signal just before the signal’s uncommon shift; X_turbRaw1_ biomass estimate from the raw turbidity sensor signal immediately after the signal uncommon shift; diff. in % (parameter in parentheses) = ABS(X_turbRaw1_ − X_turbRaw0_)*100%/X_turbRaw0_; for columns (6), (8) and (9) analogic description as for column (5) using appropriate parameters of X_turbFiltr_, X_permRaw_ and X_permFiltr_; (7) maximum RSn of the permittivity signal reached in approximately 2 to 3 min starting from time indicated in column 1.

## Appendix C

Volume of gas mixing chamber ≈1 L.

## Appendix D

Exhaust gass filter cloging happen in experiment 7s. This may explain bad *X_OTR_* and *X_CPR_* estimation results in experiment 7s.
